# Supplementary material for: Medication-Related Hospital Admissions and Emergency Department Visits in Older People with Diabetes: A Systematic Review
Source: J Clin Med. 2024 Jan 17;13(2):530. doi: 10.3390/jcm13020530 (PMC10817070; doi:10.3390/jcm13020530)
Supplement: Supplementary file 1 [file jcm-13-00530-s001.zip › jcm-2792778-supplementary.pdf]

Supplementary Materials

Table S1: Embase Search Strategies and Results.

Embase <1974 to October 20, 2023>

Search Number	Search term	Results
1	side effect/	369,934
2	adverse drug reaction/	285,942
3	medication error/	20,682
4	Inappropriate Prescribing/	5,056
5	Polypharmacy/	24,058
6	Adverse Reaction*.ti,ab,kf.	69,748
7	medication error*.ti,ab,kf.	12,269
8	((medication adj2 related) or (medication adj2 problem*)).ti,ab,kf.	11,572
9	((adverse adj2 reaction*) or (adverse adj2 event*)).ti,ab,kf.	548,929
10	((drug adj2 related) or (drug adj2 problem*)).ti,ab,kf.	51,900
11	((medicine adj2 related) or (medicine adj2 problem*)).ti,ab,kf.	2,878
12	potentially inappropriate prescription*.ti,ab,kf.	536
13	potentially inappropriate medication*.ti,ab,kf.	2,795
14	drug induced.ti,ab,kf.	65,175
15	Hypoglycemia/	94,258
16	Hypoglyc*mi*.ti,ab,kf.	102,460
17	1 or 2 or 3 or 4 or 5 or 6 or 7 or 8 or 9 or 10 or 11 or 12 or 13 or 14 or 15 or 16	1,244,668
18	exp antidiabetic agent/	599,719
19	dipeptidyl peptidase IV inhibitor/	14,197
20	exp sulfonylurea/	18,620
21	acarbose/	10,585
22	exp glucagon like peptide 1 receptor agonist/	48,975
23	insulin.ti,ab,kf.	557,369
24	Metformin.ti,ab,kf.	48,086
25	Sul*onylurea*.ti,ab,kf.	17,166
26	GLP*1RA.ti,ab,kf.	221
27	SGLT2* inhibitor*.ti,ab,kf.	8,522
28	DPP* inhibitor*.ti,ab,kf.	1,840
29	18 or 19 or 20 or 21 or 22 or 23 or 24 or 25 or 26 or 27 or 28	848,769
30	hospitalization/	535,491
31	Emergency Service, Hospital/	9,419
32	hospital admission*.ti,ab,kf.	87,571
33	hospitali*.ti,ab,kf.	566,526
34	admission*.ti,ab,kf.	508,450

35	admitted.ti,ab,kf.	472,800
36	(emergency adj3 visit*).ti,ab,kf.	40,048
37	(emergency adj3 service*).ti,ab,kf.	37,144
38	acute care.ti,ab,kf.	40,720
39	30 or 31 or 32 or 33 or 34 or 35 or 36 or 37 or 38	1,534,481
40	diabetes mellitus/	706,723
41	diabetes.ti,ab,kf.	1,031,261
42	T2D*.ti,ab,kf.	87,708
43	T1D*.ti,ab,kf.	34,264
44	40 or 41 or 42 or 43	1,260,211
45	17 and 29 and 39 and 44	8,934
46	limit 45 to (english language and yr="2000 -Current")	8,160
47	46 not ((exp animal/ or exp invertebrate/ or nonhuman/ or animal experiment/ or animal tissue/ or animal model/ or exp plant/ or exp fungus/) not (exp human/ or human tissue/))	8,126
48	limit 47 to "remove medline records"	4,816
49	case report/	2,926,648
50	conference paper/	765,635
51	editorial/	751,223
52	practice guideline/	561,423
53	newborn/	605,820
54	child/	2,115,397
55	clinical trial/ or randomized controlled trial/ or controlled clinical trial/	1,579,385
56	conference review/ or "peer review"/ or "peer review organization"/ or "systematic review (topic)"/ or "medical record review"/ or "professional standards review organization"/ or "institutional review"/ or "systematic review"/ or "drug utilization review"/ or "utilization review"/ or "review"/	3,475,930
57	49 or 50 or 51 or 52 or 53 or 54 or 55 or 56	11,337,281
58	48 not 57	2,483

Table S2: Medline Search Strategies and Results.

Ovid MEDLINE(R) ALL <1946 to October 20, 2023>

Search Number	Search term	Results
1	Drug-Related Side Effects and Adverse Reactions/	38,520
2	Medication Errors/	14,381
3	Inappropriate Prescribing/	4,638
4	Polypharmacy/	6,742
5	Adverse Reaction*.ti,ab,kf.	44,035
6	medication error*.ti,ab,kf.	7,122

7	((medication adj2 related) or (medication adj2 problem*)).ti,ab,kf.	7,423
8	((adverse adj2 reaction*) or (adverse adj2 event*)).ti,ab,kf.	317,654
9	((drug adj2 related) or (drug adj2 problem*)).ti,ab,kf.	33,132
10	((medicine adj2 related) or (medicine adj2 problem*)).ti,ab,kf.	2,229
11	potentially inappropriate prescription*.ti,ab,kf.	334
12	potentially inappropriate medication*.ti,ab,kf.	1,770
13	drug induced.ti,ab,kf.	47,365
14	Hypoglycemia/	30,601
15	Hypoglyc*mi*.ti,ab,kf.	67,843
16	1 or 2 or 3 or 4 or 5 or 6 or 7 or 8 or 9 or 10 or 11 or 12 or 13 or 14 or 15	509,202
17	hypoglycemic agents/ or dipeptidyl-peptidase iv inhibitors/ or sodium-glucose transporter 2 inhibitors/	83,646
18	Insulin/	199,748
19	Metformin/	18,047
20	Sulfonylurea Compounds/	6,682
21	Acarbose/	1,579
22	insulin.ti,ab,kf.	411,646
23	GLP-1 Receptor Agonists.ti,ab,kf.	1,934
24	Dipeptidyl-Peptidase IV Inhibitors.ti,ab,kf.	414
25	DPP* inhibitor*.ti,ab,kf.	953
26	Metformin.ti,ab,kf.	28,340
27	Sul*onylurea*.ti,ab,kf.	11,523
28	17 or 18 or 19 or 20 or 21 or 22 or 23 or 24 or 25 or 26 or 27	505,820
29	Hospitalization/	136,495
30	Emergency Service, Hospital/	87,989
31	hospital admission*.ti,ab,kf.	52,655
32	hospitali*.ti,ab,kf.	348,243
33	admission*.ti,ab,kf.	290,480
34	admitted.ti,ab,kf.	265,231
35	(emergency adj3 visit*).ti,ab,kf.	25,354
36	(emergency adj3 service*).ti,ab,kf.	26,478
37	acute care.ti,ab,kf.	28,640
38	29 or 30 or 31 or 32 or 33 or 34 or 35 or 36 or 37	889,838
39	Diabetes Mellitus/	140,079
40	diabetes.ti,ab,kf.	670,839
41	T2D*.ti,ab,kf.	50,816
42	T1D*.ti,ab,kf.	17,047
43	39 or 40 or 41 or 42	709,153
44	16 and 28 and 38 and 43	2,770
45	limit 44 to (english language and yr="2000 -Current")	2,484
46	45 not (exp animals/ not humans.sh.)	2,479
47	Randomized Controlled Trial/ or Clinical Trial/	924,068

48	Utilization Review/ or Peer Review, Research/ or "Scientific Integrity Review"/ or "Systematic Review"/ or "Review"/ or review.mp. or "Rate Setting and Review"/ or "Peer Review"/ or "Ethical Review"/ or "Drug Utilization Review"/ or "Concurrent Review"/ or "Professional Review Organizations"/ or "Physician Payment Review Commission"/ or "Review Literature as Topic"/ or "Insurance Claim Review"/ or Peer Review, Health Care/ or "Medication Review"/	4,070,722
49	Practice Guideline/ or Guideline/	37,994
50	Case Reports/	2,361,817
51	Editorial/	668,257
52	Infant, Newborn/	672,771
53	Child/	1,926,360
54	47 or 48 or 49 or 50 or 51 or 52 or 53	9,520,228
55	46 not 54	1,257

Table S3: International Pharmaceutical Abstracts Search Strategies and Results.

International Pharmaceutical Abstracts <1970 to January 6, 2023>

Search Number	Search term	Results
1	Drug-Related Side Effects.mp. [mp=title, subject heading word, registry word, abstract, trade name/generic name]	88
2	Adverse Reactions.mp. [mp=title, subject heading word, registry word, abstract, trade name/generic name]	23,277
3	1 or 2	23,357
4	Medication Errors.mp. [mp=title, subject heading word, registry word, abstract, trade name/generic name]	3,333
5	Inappropriate Prescribing.mp. [mp=title, subject heading word, registry word, abstract, trade name/generic name]	402
6	Polypharmacy.mp. [mp=title, subject heading word, registry word, abstract, trade name/generic name]	1,538
7	Adverse Reaction*.ti,ab.	7,303
8	medication error*.ti,ab.	3,693
9	((medication adj2 related) or (medication adj2 problem*)).ti,ab.	1,718
10	((adverse adj2 reaction*) or (adverse adj2 event*)).ti,ab.	32,059
11	((drug adj2 related) or (drug adj2 problem*)).ti,ab.	4,936
12	((medicine adj2 related) or (medicine adj2 problem*)).ti,ab.	107
13	potentially inappropriate prescription*.ti,ab.	47
14	potentially inappropriate medication*.ti,ab.	286
15	drug induced.ti,ab.	3,083
16	Hypoglycemia.mp. [mp=title, subject heading word, registry word, abstract, trade name/generic name]	2,790
17	Hypoglyc*mi*.ti,ab.	5,303

18	3 or 4 or 5 or 6 or 7 or 8 or 9 or 10 or 11 or 12 or 13 or 14 or 15 or 16 or 17	61,075
19	(hypoglycemic agents or dipeptidyl-peptidase iv inhibitors or sodium-glucose transporter 2 inhibitors).mp. [mp=title, subject heading word, registry word, abstract, trade name/generic name]	461
20	Insulin.mp. [mp=title, subject heading word, registry word, abstract, trade name/generic name]	13,012
21	Metformin.mp. [mp=title, subject heading word, registry word, abstract, trade name/generic name]	3,675
22	Sulfonylurea Compounds.mp. [mp=title, subject heading word, registry word, abstract, trade name/generic name]	3
23	Acarbose.mp. [mp=title, subject heading word, registry word, abstract, trade name/generic name]	458
24	insulin.ti,ab.	12,120
25	GLP-1 Receptor Agonists.ti,ab.	162
26	Dipeptidyl-Peptidase IV Inhibitors.ti,ab.	38
27	DPP* inhibitor*.ti,ab.	40
28	Metformin.ti,ab.	3,041
29	Sul*onylurea*.ti,ab.	1,330
30	19 or 20 or 21 or 22 or 23 or 24 or 25 or 26 or 27 or 28 or 29	16,175
31	Hospitalization.mp. [mp=title, subject heading word, registry word, abstract, trade name/generic name]	6,127
32	Emergency Service.mp. [mp=title, subject heading word, registry word, abstract, trade name/generic name]	43
33	hospital admission*.ti,ab.	2,026
34	hospitali*.ti,ab.	12,986
35	admission*.ti,ab.	7,859
36	admitted.ti,ab.	6,066
37	(emergency adj3 visit*).ti,ab.	1,177
38	(emergency adj3 service*).ti,ab.	330
39	"acute care".ti,ab.	1,118
40	31 or 32 or 33 or 34 or 35 or 36 or 37 or 38 or 39	23,847
41	Diabetes Mellitus.mp. [mp=title, subject heading word, registry word, abstract, trade name/generic name]	18,050
42	diabetes.ti,ab.	19,992
43	T2D*.ti,ab.	1,516
44	T1D*.ti,ab.	196
45	41 or 42 or 43 or 44	22,959
46	18 and 30 and 40 and 45	313
47	limit 46 to (english language and yr="2000 -Current")	266

Table S4: Summary of the quality assessment for cross-sectional studies (n = 16).

Checklist for prevalence studies	Sotiropoulos et al (2005) [1]	Feil et al (2011) [2]	Salutini et al (2015) [3]	Kim et al (2016) [4]	Mantovani et al (2016) [5]	Conceição et al (2017) [6]	Mazzi et al (2017) [7]	Kaewput et al (2019) [8]	Müller et al (2020) [9]	Lee et al (2021) [10]	Nuzzo et al (2022) [11]	Chen et al (2022)[12]	Galea et al (2022) [13]	Galindoet al (2022) [14]	Yun et al (2022) [15]	Horii et al (2023) [16]
Were the criteria for inclusion in the sample clearly defined?	Yes	Yes	Yes	Yes	No	Yes	Yes	Yes	Yes	Yes	Yes	Yes	Yes	Yes	Yes	Yes
Were the study subjects and the setting described in detail?	Yes	Yes	Yes	Yes	Yes	Yes	Yes	Yes	Yes	Yes	Yes	Yes	Yes	Yes	Yes	Yes
Was the exposure measured in a valid and reliable way?	Unclear	Yes	No	Yes	Yes	Yes	Yes	Yes	Yes	Yes	Yes	Yes	Yes	Yes	Yes	Yes
Were objective, standard criteria used for measurement of the condition?	Yes	Yes	Yes	Yes	Not applicable	Yes	No	Yes	Yes	Yes	Yes	Yes	Yes	Yes	Yes	Yes
Were confounding factors identified?	No	Yes	No	Yes	No	Yes	No	Yes	Yes	Yes	Yes	No	No	Yes	Yes	Yes
Were strategies to deal with confounding factors stated?	No	Yes	No	Yes	No	No	No	No	No	Yes	Yes	No	No	Yes	No	No
Were the outcomes measured in a valid and reliable way?	Yes	Yes	Yes	Yes	Yes	Yes	Yes	Yes	Yes	Yes	Yes	Yes	Yes	Yes	Yes	Yes
Was appropriate statistical analysis used?	Unclear	Yes	Yes	Yes	Yes	Yes	Yes	Yes	Yes	Yes	Yes	Yes	No	Yes	Yes	Yes
	Yes=4;Unclear=2; No=2	Yes=8	Yes=5; No=3	Yes=8	Yes=4;Not applicable=1 ; No=3	Yes=7; No=1	Yes=5; No=3	Yes=7; No=1	Yes=7; No=1	Yes=8	Yes=8	Yes=6; No=2	Yes=5; No=3	Yes=8	Yes=7; No=1	Yes=7; No=1
Study quality	Moderate	High	Moderate	High	Moderate	High	Moderate	High	High	High	High	Moderate	Moderate	High	High	High

Scores can range from 0 to 8, each question is given a single score if the answer is Yes

Study quality: High quality: score ≥6, Moderate quality: score 4-5, Low quality: score ≤3

Table S5: Summary of the quality assessment for cohort studies (n = 10).

Checklist for prevalence studies	Tschöpe et al (2012) [17]	Fu et al (2014) [18]	Hung et al (2017) [19]	Zhong et al (2017) [20]	Misra-Hebert et al (2018) [21]	Andreano et al (2020) [22]	McCoy et al (2020) [23]	Jensen et al (2021) [24]	Lacy et al (2021) [25]	Pilla et al (2021) [26]
Were the two groups similar and recruited from the same population?	Not applicable	Not applicable	Yes	Not applicable	Not applicable	Not applicable	Not applicable	Not applicable	Not applicable	Not applicable
Were the exposures measured similarly to assign people to both exposed and unexposed groups?	Not applicable	Not applicable	Yes	Not applicable	Not applicable	Not applicable	Not applicable	Not applicable	Not applicable	Not applicable
Was the exposure measured in a valid and reliable way?	Yes	Yes	Yes	Yes	Yes	Yes	Yes	Yes	Yes	No
Were confounding factors identified?	Yes	No	No	No	Yes	Yes	Yes	Yes	No	Yes
Were strategies to deal with confounding factors stated?	No	No	No	No	Yes	Yes	Yes	No	No	No
Were the groups/participants free of the outcome at the start of the study (or at the moment of exposure)?	Yes	Yes	Yes	Yes	Yes	Yes	Yes	Yes	Yes	Yes
Were the outcomes measured in a valid and reliable way?	No	Yes	Yes	Yes	Yes	Yes	Yes	Yes	Yes	Yes
Was the follow up time reported and sufficient to be long enough for outcomes to occur?	Yes	Yes	Yes	Yes	Yes	Yes	Yes	Yes	Yes	Yes
Was follow up complete, and if not, were the reasons to loss to	Yes	Yes	Yes	Yes	Yes	Yes	Yes	Yes	Yes	Yes

follow up described and explored?										
Were strategies to address incomplete follow up utilized?	Yes	Yes	Not applicable	Yes	No	Yes	No	Yes	Yes	Yes
Was appropriate statistical analysis used?	Yes	Yes	Yes	Yes	Yes	Yes	No	Yes	Yes	Yes
	Yes=7;Not applicable=2; No=2	Yes=7;Not applicable=2; No=2	Yes=8;Not applicable=1; No=2	Yes=7;Not applicable=2; No=2	Yes=8;Not applicable=2; No=1	Yes=9;Not applicable=2	Yes=7;Not applicable=2; No=2	Yes=7;Not applicable=2; No=2	Yes=8;Not applicable=2; No=1	Yes=7;Not applicable=2; No=2
Study quality	Moderate	Moderate	High	Moderate	High	High	Moderate	Moderate	High	Moderate

Scores can range from 0 to 11, each question is given a single score if the answer is Yes

Study quality: High quality: score ≥8, Moderate quality: score 5-7, Low quality: score ≤4

Table S6: Summary of the quality assessment for prevalence studies (n = 6).

Checklist for prevalence studies	Greco et al (2010) [27]	Nazish et al (2014) [28]	Namba et al (2018) [29]	Caballero-Corchuelo et al (2019) [30]	Pereira et al (2020) [31]	Poret et al (2022) [32]
Was the sample frame appropriate to address the target population?	Yes	Yes	Yes	Yes	Yes	Yes
Were study participants sampled in an appropriate way?	Yes	Yes	Yes	Yes	Yes	Yes
Was the sample size adequate?	Yes	Yes	Yes	Yes	Yes	Yes
Were the study subjects and the setting described in detail?	Yes	No	Yes	Yes	No	Yes
Was the data analysis conducted with sufficient coverage of the identified sample?	Unclear	Yes	Yes	Yes	Yes	Yes
Were valid methods used for the identification of the condition?	Yes	No	Yes	Yes	Yes	Yes
Was the condition measured in a standard, reliable way for all participants?	Yes	No	Yes	Yes	Yes	Yes
Was there appropriate statistical analysis?	Yes	No	Yes	Yes	No	Yes
Was the response rate adequate, and if not, was the low response rate managed appropriately?	Yes	Yes	Yes	Yes	Yes	Yes
	Yes=8; Unclear 1	Yes=5; No=4	Yes=9	Yes=9	Yes=7;No=2	Yes=9
Study quality	High	Moderate	High	High	High	High

Scores can range from 0 to 9, each question is given a single score if the answer is Yes

Study quality: High quality: score ≥7, Moderate quality: score 4-6, Low quality: score ≤3

Supplementary Table S7: Summary of the quality assessment for case-control studies (n = 3).

**Checklist for prevalence studies**	**Liatis et al (2015)[33]**	**Park et al (2018)[34]**	**Bengtsen et al (2021)[35]**
Were the groups comparable other than the presence of disease in cases or the absence of disease in controls?	No	Yes	Yes
Were cases and controls matched appropriately?	No	Yes	Yes
Were the same criteria used for identification of cases and controls?	No	Yes	Yes
Was exposure measured in a standard, valid and reliable way?	Yes	Yes	Yes
Was exposure measured in the same way for cases and controls?	Yes	Yes	Yes
Were confounding factors identified?	No	No	No
Were strategies to deal with confounding factors stated?	No	No	No
Were outcomes assessed in a standard, valid and reliable way for cases and controls?	Yes	Yes	Yes
Was the exposure period of interest long enough to be meaningful?	Yes	Yes	Yes
Was appropriate statistical analysis used?	Yes	Yes	Yes
	Yes=5; No=5	Yes=8; No=2	Yes=8; No=2
Study quality	Moderate	High	High

Scores can range from 0 to 10, each question is given a single score if the answer is Yes

Study quality: High quality: score ≥7, Moderate quality: score 5-6, Low quality: score ≤4

References

1. Sotiropoulos, A.; Skliros, E.; Tountas, C.; Apostolou, U.; Peppas, T.; Pappas, S. Risk factors for severe hypoglycaemia in type 2 diabetic patients admitted to hospital in Piraeus, Greece. *EMHJ-East. Mediterr. Health J.* **2005**, *11*, 485-489.
1. Feil, D.G.; Rajan, M.; Soroka, O.; Tseng, C.-L.; Miller, D.R.; Pogach, L.M. Risk of hypoglycemia in older veterans with dementia and cognitive impairment: implications for practice and policy. *J. Am. Geriatr. Soc.* **2011**, *59*, 2263-2272.
2. Salutini, E.; Bianchi, C.; Santini, M.; Dardano, A.; Daniele, G.; Penno, G.; Miccoli, R.; Del Prato, S. Access to emergency room for hypoglycaemia in people with diabetes. *Diabetes Metab Res Rev* **2015**, *31*, 745-751. https://doi.org/10.1002/dmrr.2667.
3. Kim, H.M.; Seong, J.M.; Kim, J. Risk of hospitalization for hypoglycemia among older Korean people with diabetes mellitus: Interactions between treatment modalities and comorbidities. *Med. (Baltim.)* **2016**, *95*, e5016.
4. Mantovani, A.; Grani, G.; Chioma, L.; Vancieri, G.; Giordani, I.; Rendina, R.; Rinaldi, M.E.; Andreadi, A.; Coccaro, C.; Boccardo, C. Severe hypoglycemia in patients with known diabetes requiring emergency department care: A report from an Italian multicenter study. *J. Clin. Transl. Endocrinol.* **2016**, *5*, 46-52.
5. Conceição, J.; Dores, J.; Araújo, F.; Laires, P.A.; Carr, R.D.; Brodovicz, K.; Radican, L.; Nogueira, A.M. Severe hypoglycaemia among patients with type 2 diabetes requiring emergency hospital admission: T he H ypoglycaemia I n P ortugal O bservational S tudy–E mergency R oom (HIPOS–ER). *Diabetes Obes. Metab.* **2018**, *20*, 50-59.
6. Mazzi, S.; Ravasio, R.; Forlani, G.; Veronese, G.; Fabbri, A.; Marchesini, G. Estimating the risk of severe hypoglycemic event related to glucose-lowering treatment among Italian patients with diabetes: the HYPOTHESIS database. *Clin. Outcomes Res.* **2017**, 711-720.
7. Kaewput, W.; Thongprayoon, C.; Varothai, N.; Sirirungreung, A.; Rangsin, R.; Bathini, T.; Mao, M.A.; Cheungpasitporn, W. Prevalence and associated factors of hospitalization for dysglycemia among elderly type 2 diabetes patients: A nationwide study. *World J. Diabetes* **2019**, *10*, 212.
8. Müller, N.; Lehmann, T.; Klöss, A.; Günster, C.; Kloos, C.; Müller, U.A. Changes in incidence of severe hypoglycaemia in people with type 2 diabetes from 2006 to 2016: analysis based on health insurance data in Germany considering the anti‐hyperglycaemic medication. *Diabet. Med.* **2020**, *37*, 1326-1332.
9. Lee, S.E.; Kim, K.-A.; Son, K.J.; Song, S.O.; Park, K.H.; Park, S.H.; Nam, J.Y. Trends and risk factors in severe hypoglycemia among individuals with type 2 diabetes in Korea. *Diabetes Res. Clin. Pract.* **2021**, *178*, 108946.
10. Nuzzo, A.; Brignoli, A.; Ponziani, M.C.; Zavattaro, M.; Prodam, F.; Castello, L.M.; Avanzi, G.C.; Marzullo, P.; Aimaretti, G. Aging and comorbidities influence the risk of hospitalization and mortality in diabetic patients experiencing severe hypoglycemia. *Nutr. Metab. Cardiovasc. Dis.* **2022**, *32*, 160-166.
11. Chen, N.-C.; Chen, C.-L.; Shen, F.-C. The Risk Factors of Severe Hypoglycemia in Older Patients with Dementia and Type 2 Diabetes Mellitus. *J. Pers. Med.* **2022**, *12*, 67.
12. Galea, A.; Farrugia, S.; Calleja, N.; Coppini, D.V. Risk factors and outcomes of severe hypoglycaemia requiring hospital admission: a retrospective study. *Pract. Diabetes* **2022**, *39*, 19.
13. Galindo, R.J.; Ali, M.K.; Funni, S.A.; Dodge, A.B.; Kurani, S.S.; Shah, N.D.; Umpierrez, G.E.; McCoy, R.G. Hypoglycemic and hyperglycemic crises among US adults with diabetes and end-stage kidney disease: Population-based study, 2013–2017. *Diabetes Care* **2022**, *45*, 100-107.
14. Yun, J.S.; Han, K.; Ko, S.H. Trends of severe hypoglycemia in patients with type 2 diabetes in Korea: A longitudinal nationwide cohort study. *J. Diabetes Investig.* **2022**, *13*, 1438-1443.
15. Horii, T.; Otsuka, M.; Yasu, T. Risk of non-hypoglycemic agents for hypoglycemia-related hospitalization in patients with type 2 diabetes: a large-scale medical receipt database analysis. *BMJ Open Diabetes Res. Care* **2023**, *11*. https://doi.org/10.1136/bmjdrc-2022-003177.
16. Tschöpe, D.; Bramlage, P.; Binz, C.; Krekler, M.; Deeg, E.; Gitt, A.K. Incidence and predictors of hypoglycaemia in type 2 diabetes–an analysis of the prospective DiaRegis registry. *BMC Endocr. Disord.* **2012**, *12*, 1-9.
17. Fu, H.; Xie, W.; Curtis, B.; Schuster, D. Identifying factors associated with hypoglycemia-related hospitalizations among elderly patients with T2DM in the US: a novel approach using influential variable analysis. *Curr. Med. Res. Opin.* **2014**, *30*, 1787-1793.
18. Hung, Y.; Lin, C.; Chen, H.; Chang, M.; Huang, K.; Chen, Y.; Chen, C. Severe hypoglycemia and hip fracture in patients with type 2 diabetes: a nationwide population-based cohort study. *Osteoporos. Int.* **2017**, *28*, 2053-2060.
19. Zhong, V.W.; Juhaeri, J.; Cole, S.R.; Kontopantelis, E.; Shay, C.M.; Gordon-Larsen, P.; Mayer-Davis, E.J. Incidence and trends in hypoglycemia hospitalization in adults with type 1 and type 2 diabetes in England, 1998–2013: a retrospective cohort study. *Diabetes Care* **2017**, *40*, 1651-1660.
20. Misra-Hebert, A.D.; Pantalone, K.M.; Ji, X.; Milinovich, A.; Dey, T.; Chagin, K.M.; Bauman, J.M.; Kattan, M.W.; Zimmerman, R.S. Patient characteristics associated with severe hypoglycemia in a type 2 diabetes cohort in a large, integrated health care system from 2006 to 2015. *Diabetes Care* **2018**, *41*, 1164-1171.
21. Andreano, A.; Bosio, M.; Russo, A.G. Emergency attendance for acute hyper-and hypoglycaemia in the adult diabetic population of the metropolitan area of Milan: quantifying the phenomenon and studying its predictors. *BMC Endocr. Disord.* **2020**, *20*, 1-14.
22. McCoy, R.G.; Lipska, K.J.; Van Houten, H.K.; Shah, N.D. Association of cumulative multimorbidity, glycemic control, and medication use with hypoglycemia-related emergency department visits and hospitalizations among adults with diabetes. *JAMA Netw. Open* **2020**, *3*, e1919099-e1919099.

23. Jensen, M.H.; Hejlesen, O.; Vestergaard, P. Epidemiology of hypoglycaemic episodes leading to hospitalisations in Denmark in 1998–2018. *Diabetologia* **2021**, *64*, 2193-2203.
24. Lacy, M.E.; Whitmer, R.A.; Lee, S.J.; Rushakoff, R.J.; Pletcher, M.J. Identifying Potential Intervention Points for Acute Hypoglycemic Events in Patients With Type 2 Diabetes Using Retrospective Clinical Data. *Clin. Diabetes* **2021**, *39*, 304-312.
25. Pilla, S.J.; Kraschnewski, J.L.; Lehman, E.B.; Kong, L.; Francis, E.; Poger, J.M.; Bryce, C.L.; Maruthur, N.M.; Yeh, H.-C. Hospital utilization for hypoglycemia among patients with type 2 diabetes using pooled data from six health systems. *BMJ Open Diabetes Res. Care* **2021**, *9*, e002153.
26. Greco, D.; Pisciotta, M.; Gambina, F.; Maggio, F. Severe hypoglycaemia leading to hospital admission in type 2 diabetic patients aged 80 years or older. *Exp. Clin. Endocrinol. Diabetes* **2010**, *118*, 215-219.
27. Nazish, Z.; Inayatullah, M.; Mustafa, F. Risk factors of hypoglycemia in diabetics. In Proceedings of the Medical Forum Monthly, 2014; pp. 38-42.
28. Namba, M.; Iwakura, T.; Nishimura, R.; Akazawa, K.; Matsuhisa, M.; Atsumi, Y.; Satoh, J.; Yamauchi, T. The current status of treatment-related severe hypoglycemia in Japanese patients with diabetes mellitus: A report from the committee on a survey of severe hypoglycemia in the Japan Diabetes Society. *J. Diabetes Investig.* **2018**, *9*, 642-656.
29. Caballero-Corchuelo, J.; Guerrero-Perez, F.; Garcia-Sancho de la Jordana, P.; Perez-Maraver, M. Analysis of the characteristics of patients with diabetes mellitus who attend a tertiary hospital emergency department for a hypoglycemic event. *Endocrinol Diabetes Nutr (Engl Ed)* **2019**, *66*, 19-25.
30. Pereira, C.A.; Almeida, R.; Dores, J. Characteristics of hypoglycaemic episodes in diabetic patients treated at the emergency room of a Portuguese tertiary centre, 2012 - 2016. *Endocrinol. Diabetes Metab.* **2020**, *3*, e00150.
31. Poret, F.; Nacher, M.; Pujo, J.; Cauvin, J.M.; Demar, M.; Massicard, M.; Sabbah, N. Risk factors for hypoglycaemia in people with diabetes admitted to the Emergency Department of a Hospital in French Guiana. *Diabet. Med.* **2022**, *39*, e14736.
32. Liatis, S.; Mylona, M.; Kalopita, S.; Papazafiropoulou, A.; Karamagkiolis, S.; Melidonis, A.; Xilomenos, A.; Ioannidis, I.; Kaltsas, G.; Lanaras, L. Hypoglycaemia requiring medical assistance in patients with diabetes: a prospective multicentre survey in tertiary hospitals. *Diabetes Metab.* **2015**, *41*, 126-131.
33. Park, S.-Y.; Jang, E.J.; Shin, J.-Y.; Lee, M.-Y.; Kim, D.; Lee, E.-K. Prevalence and predictors of hypoglycemia in South Korea. *Am J Manag Care* **2018**, *24*, 278-286.
34. Bengtsen, M.B.; Knudsen, J.S.; Bengtsen, M.B.; Møller, N.; Thomsen, R.W. Hospitalization for hypoglycaemia in people with diabetes in Denmark, 1997 - 2017: Time trends in incidence and HbA1c and glucose - lowering drug use before and after hypoglycaemia. *Endocrinol. Diabetes Metab.* **2021**, *4*, e00227.

Disclaimer/Publisher's Note: The statements, opinions and data contained in all publications are solely those of the individual author(s) and contributor(s) and not of MDPI and/or the editor(s). MDPI and/or the editor(s) disclaim responsibility for any injury to people or property resulting from any ideas, methods, instructions or products referred to in the content.
